# Headache Over Heels: CT Negative Subarachnoid Hemorrhage

**DOI:** 10.21980/J8ND2C

**Published:** 2023-07-31

**Authors:** Sarah Hogan, Sara Dimeo, Caroline Astemborski

**Affiliations:** * Prisma Health Upstate, University of South Carolina School of Medicine Greenville, Department of Emergency Medicine, Greenville, SC; ^ Dignity Health East Valley, Department of Emergency Medicine, Chandler, AZ

## Abstract

**Audience:**

This simulation is intended for MS4 or PGY-1 learners.

**Introduction:**

Both headache and syncope are common chief complaints in the emergency department (ED); however, subarachnoid hemorrhage (SAH) is uncommon (accounting for 1–3% of all patients presenting to the ED with headache), with near 50% mortality.^1^–^3^ It is important to recognize the signs and symptoms that point to this specific diagnosis. Once subarachnoid hemorrhage is suspected, it is critical to understand the appropriate workup to diagnose SAH, depending on the timing of presentation. Once SAH is diagnosed, appropriately managing the patient’s glucose, blood pressure, and pain is important.

**Educational Objectives:**

By the end of this case, the participant will be able to: 1) construct a broad differential diagnosis for a patient presenting with syncope, 2) name the history and physical exam findings consistent with SAH, 3) identify SAH on computer tomography (CT) imaging, 4) identify the need for lumbar puncture (LP) to diagnose SAH when CT head is non-diagnostic > 6 hours after symptom onset, 5) correctly interpret cerebral fluid studies (CSF) to aid in the diagnosis of SAH, and 6) specify blood pressure goals in SAH and suggest appropriate medication management.

**Educational Methods:**

High-fidelity simulation was utilized since this modality forces learners to actively construct a differential for syncope, recognize the possibility of subarachnoid hemorrhage, recall the need for lumbar puncture, and talk through management considerations in real time as opposed to a more passive lecture format.

**Research Methods:**

Twenty emergency medicine residents and medical student learners completed the simulation activity. Each learner was asked to complete an eight question post-simulation survey. The survey addressed the utility and appropriate training level of the simulation activity while also including an open-ended prompt for suggestions for improvement.

**Results:**

Five PGY3, four PGY2, four PGY1, and seven medical students completed the survey. Ninety-five percent felt that the case was more helpful in a simulation format than in a lecture format. All learners felt that the simulation was an appropriate level of difficulty. Of the comments received, a few learners noted they preferred more complexity.

**Discussion:**

Overall, the educational content was effective in teaching about the SAH diagnostic algorithm, CSF interpretation, and blood pressure management in SAH. Overall, learners very much enjoyed the activity and felt it was appropriate for their level of training. The most common constructive feedback was to include more specific neurologic findings on physical examination to help guide the student to the diagnosis of SAH.

**Topics:**

Syncope, subarachnoid hemorrhage, cerebrospinal fluid interpretation, lumbar puncture, intracranial bleed, blood pressure goals and management.

## USER GUIDE

[Table t1-jetem-8-3-s34]

**Table t1-jetem-8-3-s34:** 

List of Resources:	
Abstract	34
User Guide	36
Instructor Materials	38
Operator Materials	48
Debriefing and Evaluation Pearls	50
Simulation Assessment	54

**Learner Audience:**

Medical Students, Interns, Junior Residents, Senior Residents

**Time Required for Implementation:**

**Instructor Preparation:** ~5 minutes

**Time for case:** ~15 minutes

**Time for debriefing:** ~10 minutes

**Recommended Number of Learners per Instructor:**

3–4

**Topics:**

Syncope, subarachnoid hemorrhage, cerebrospinal fluid interpretation, lumbar puncture, intracranial bleed, blood pressure goals and management.

**Objectives:**

By the end of this case, the participant will be able to:

1. Construct a broad differential diagnosis for a patient presenting with syncope
2. Name the history and physical exam findings consistent with SAH
3. Identify SAH on computer tomography (CT) imaging
4. Identify the need for lumbar puncture (LP) to diagnose SAH when CT head is non-diagnostic > 6 hours after symptom onset
5. Correctly interpret CSF studies to aid in the diagnosis of SAH
6. Specify blood pressure goals in SAH and suggest appropriate medication management

### Linked objectives and methods

Learners are presented with a chief complaint of “syncope” rather than “headache” to encourage them to develop a broad differential for possible causes of syncope, including neurogenic, cardiogenic, and vasovagal (objective 1). In addition, the specific history and physical exam features suggest possible SAH (objective 2), to aid the learner in including this on their differential and to demonstrate some of the classic signs and symptoms such as headache that is worst at onset, family history of aneurysm, and neck stiffness. More obvious neurologic deficits were excluded to maintain a degree of diagnostic mystery. This necessitates further history taking to differentiate between these etiologies and allows the learner to develop a pattern of questioning to use in all future syncope encounters. The patient presented outside the 6-hour time window to force the learner to consider the diagnostic algorithm for SAH as suggested by current ACEP Guidelines (published July 2019), including performing an LP (objective 4).^4^ While not included in our initial simulation, it may be beneficial to have learners perform an LP on a mannequin during this point of the case to review and practice the details of this procedure. The learner is then given the opportunity to interpret CSF studies and discuss strategies such as RBC clearance and the ratio of WBCs to RBCs, which meets objective 5.^5^, ^6^, ^7^ Lastly, to address objective 6, the patient presents with a blood pressure above the goal range of systolic pressures 140–160 mmHg allowing the learner to recognize the need for antihypertensive medications and decide on the appropriate blood pressure goals and correct antihypertensive medications.^8^

### Recommended pre-reading for instructor

Diringer MN, Bleck TP, Claude Hemphill J 3rd, et al. Critical care management of patients following aneurysmal subarachnoid hemorrhage: recommendations from the Neurocritical Care Society’s Multidisciplinary Consensus Conference. *Neurocrit Care*. 2011;15(2):211–240. doi:10.1007/s12028-011-9605-9

### Learner responsible content (optional)

Swadron S, Spangler M, Herbert M. C3 Syncope. *EM:RAP*. 2016. Available at https://www.emrap.org/c3/playlist/cardiovascular/episode/c3syncope/syncope

### Results and tips for successful implementation

Twenty emergency medicine residents and medical student learners completed the simulation activity. Each learner was asked to complete an eight question post-simulation survey, shown below. The survey addressed the utility and appropriate training level of the simulation activity while also including an open-ended prompt for suggestions for improvement. Five PGY3, four PGY2, four PGY1, and seven medical students completed the survey. Ninety-five percent felt that the case was more helpful in a simulation format than in a lecture format. All learners felt that the simulation was an appropriate level of difficulty. Of the comments received, a few learners noted they preferred more complexity.

Based on feedback from learners and facilitators, this simulation was felt to be most appropriate for the MS-4 or junior EM resident learner; however, it may benefit any EM resident. While most participants responded that the case would be best suited for an MS-4 or junior resident, all 20 respondents either agreed or strongly agreed with the statement “this case was appropriate for my level of training.” The most beneficial comments suggested more obvious neurologic deficits to aid the participant in diagnosis which is reflected in the current format of the case. Another comment called for more complexity which was incorporated by adding branch points and decompensation in mental status if the appropriate diagnostic studies are not ordered initially. After the participants obtain a thorough history and perform a physical examination, if they are not considering SAH in the diagnosis and do not ask for a CT head, the facilitator may need to draw more attention to the patient’s headache, neck pain, and neurologic deficits to guide the participant to consider this diagnosis. If CT head is still not pursued, the facilitator should point out the patient’s change in neurologic status to prompt learners to consider primary central nervous system pathology (as outlined in the timetable below).

Survey Provided to residents:

**Simulation Case: Syncope**

This case was intellectually challenging.
  Strongly Disagree – Disagree – Neutral – Agree – Strongly Agree
This case was appropriate for my level of training.
  Strongly Disagree – Disagree – Neutral – Agree – Strongly Agree
Using simulation helped me learn this topic better than a lecture would have.
  Strongly Disagree – Disagree – Neutral – Agree – Strongly Agree
The learning environment was safe and supportive.
  Strongly Disagree – Disagree – Neutral – Agree – Strongly Agree
The case facilitator helped me learn as much as possible from the case.
  Strongly Disagree – Disagree – Neutral – Agree – Strongly Agree
What would have made this session better?
[Table t2-jetem-8-3-s34]

**Table t2-jetem-8-3-s34:** 

Your Current Training level:	MS4	PGY1	PGY2	PGY3
This case would be **best** for:	MS4	PGY1	PGY2	PGY3

## Supplementary Information



## Figures and Tables

**Table t3-jetem-8-3-s34:** 

#1	Point of Care Glucose
#2	Complete Blood Count
#3	Complete Metabolic Panel
#4	Coagulation Studies
#5	Troponin
#6	D-dimer
#7	Electrocardiogram
#8	Chest X-ray
#9	Bedside Cardiac Ultrasound
#10	Non-contrast CT Head
#11	CSF Studies

**Table t4-jetem-8-3-s34:** 

**Vitals:**	Heart Rate (HR) 85	Blood Pressure (BP) 185/95
	Respiratory Rate (RR) 18	Temperature (T) 98.7°F
	Oxygen Saturation (O_2_ Sat) 98% on room air	

**Table t5-jetem-8-3-s34:** 

*Complete blood count (CBC)*	
White blood count (WBC)	4.65 **×** 1000/mm^3^
Hemoglobin (Hgb)	15.5 g/dL
Hematocrit (HCT)	47%
Platelet (Plt)	250 **×** 1000/mm^3^

**Table t6-jetem-8-3-s34:** 

*Basic metabolic panel (BMP)*	
Sodium	140 mEq/L
Potassium	4.2 mEq/L
Chloride	102 mEq/L
Bicarbonate (HCO_3_)	25 mEq/L
Blood Urea Nitrogen (BUN)	18 mg/dL
Creatinine (Cr)	1.09 mg/dL
Glucose	172 mg/dL
Calcium	9.1 mg/dL

**Table t7-jetem-8-3-s34:** 

*Troponin*	<0.01 ml/mL

**Table t8-jetem-8-3-s34:** 

*D-Dimer*	<300 ng/mL

**Table t9-jetem-8-3-s34:** 

*Coagulation Studies*	
Prothrombin time	12.5 seconds
Partial thromboplastin time	26 seconds
International normalized ratio	1.0

**Figure f1-jetem-8-3-s34:**
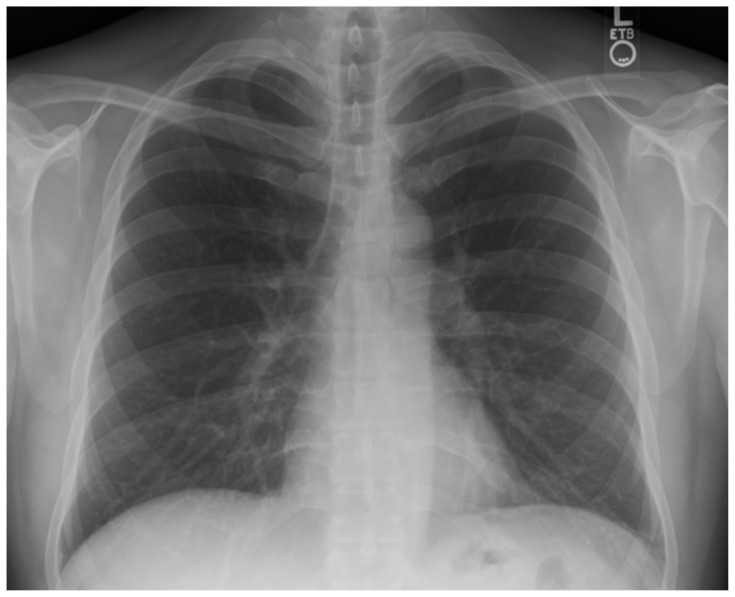


**Figure f2-jetem-8-3-s34:**
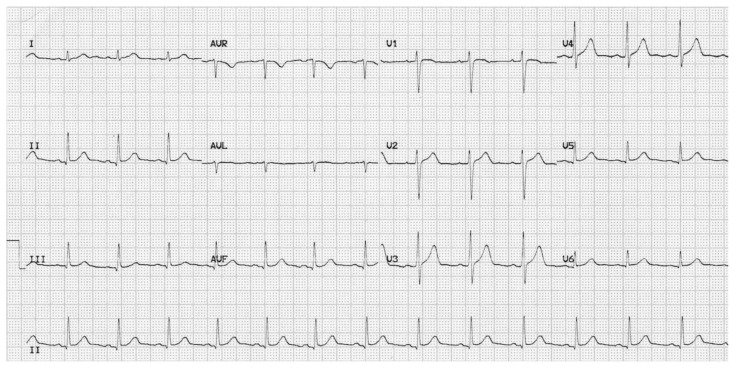


**Figure f3-jetem-8-3-s34:**
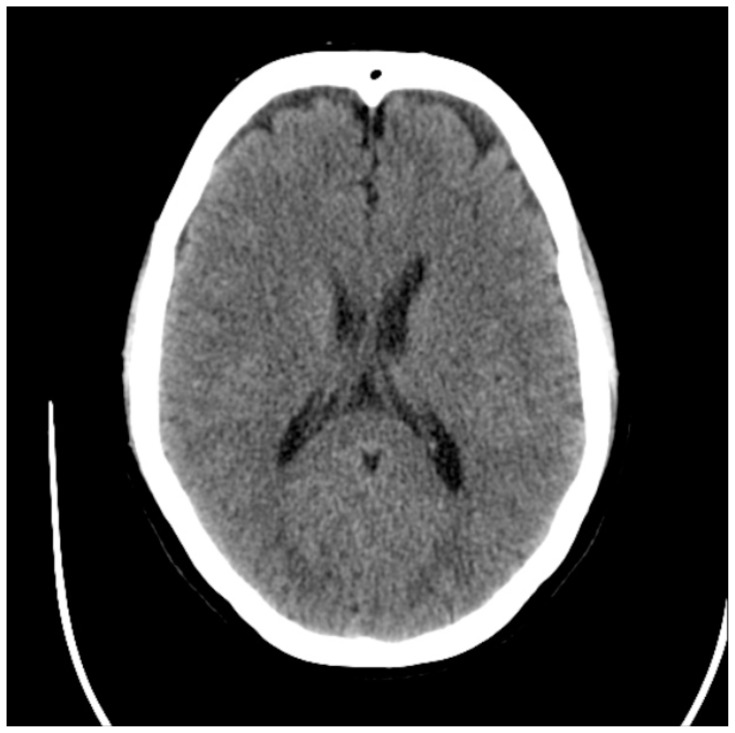


**Table t10-jetem-8-3-s34:** 

*Cerebrospinal Fluid (CSF) studies*	
Opening Pressure	25 mmHg
Appearance	grossly bloody
Protein	30 mg/dL
Glucose	115 mg/dL
White blood cells	20 WBCs/uL
RBCs in tube 1	21,115 RBCs/uL
RBCs in tube 2	21,032 RBCs/uL
RBCs in tube 3	20,876 RBCs/uL
RBCs in tube 4	20,655 RBCs/uL

**Table t11-jetem-8-3-s34:** SIMULATION EVENTS TABLE:

Minute (state)	Participant action/trigger	Patient status (simulator response) & operator prompts	Monitor display (vital signs)
0:00 (Baseline)	Place patient on monitors.		T 98.7° F HR 85 BP 185/95 RR 18 O2 98%
2:00	Obtain history and physical exam.	The patient is uncomfortable but able to answer all historical questions and participate in physical exam. If no neurologic exam performed, patient should complain of headache and neck pain again.	T 98.7° F HR 85 BP 185/95 RR 18 O2 98%
4:00	Order diagnostic studies.	If learner does not order CT head, operator should ask, “Are there any tests you would like to order to workup the patient’s headache?” or, “What do you make of the patient’s headache?”	T 98.7° F HR 85 BP 185/95 RR 18 O2 98%
6:00 If Head CT ordered.	Head CT Ordered. Interpret CT head.	CT head should be interpreted as normal without intracranial hemorrhage. Learner should then suggest lumbar puncture. If not, nurse should ask, “If you are worried about a SAH but CT head is negative, is there another study you can pursue?”	T 98.7° F HR 95 BP 190/95 RR 18 O2 98%
6:00 If LP ordered.	LP ordered. Participants obtain LP.	Participants discuss need for LP with patient, including risks, benefits, and alternatives. Patient consents. If patient has decompensated at this time, then attempt should be made to obtain consent from family.	T 98.7° F HR 95 BP 190/95 RR 18 O2 98%
10:00 If CTA is ordered.	CTA Ordered. Ask for CTA.	Facilitator verbally relays the radiology report of “ruptured aneurysm at the origin of the left anterior cerebral artery and M1 segment of left middle cerebral artery.”	T 98.7° F HR 95 BP 190/95 RR 18 O2 98%
10:00 If no CT head, CTA or LP is ordered.	If no CTA or LP is ordered after the normal CT head e	Patient complains of worsening headache and neck pain. States, “my head hurts so badly!” and then becomes less responsive. Opens eyes to command, is confused, localizes to pain (GCS 3, 4, 5 =12). Repeat Head CT ordered, Radiology calls and states large SAH.	T 98.7° F HR 105 BP 195/105 RR 18 O2 98%
12:00 If LP is obtained.	Interpret CSF	CSF should be interpreted as consistent with SAH. If the CSF is not interpreted as SAH, then lab will call and report xanthochromia as a “critical result.”	T 98.7° F HR 105 BP 195/105 RR 18 O2 98%
13:00	Once SAH is diagnosed, learners should recognize need for BP control.	The learner should reassess blood pressure and initiate IV antihypertensive to lower to goal of systolic blood pressure 140–160.	BP 192/90 HR 105 RR 18 O2 98%
15:00	Disposition.	Learner should page the Neurology Critical Care Service or Neuroendovascular Service for admission. If learner fails to consult teams for admission, nurse should state, “I’ve never seen this before; who admits this case in the hospital?” If learners consult an inappropriate service, consultant should state, “I would like to help, but I’m not the right person, I think you are looking for Neuro Critical Care.”	BP improved 160/75 HR 95 RR 18 O2 98%

**Figure f4-jetem-8-3-s34:**
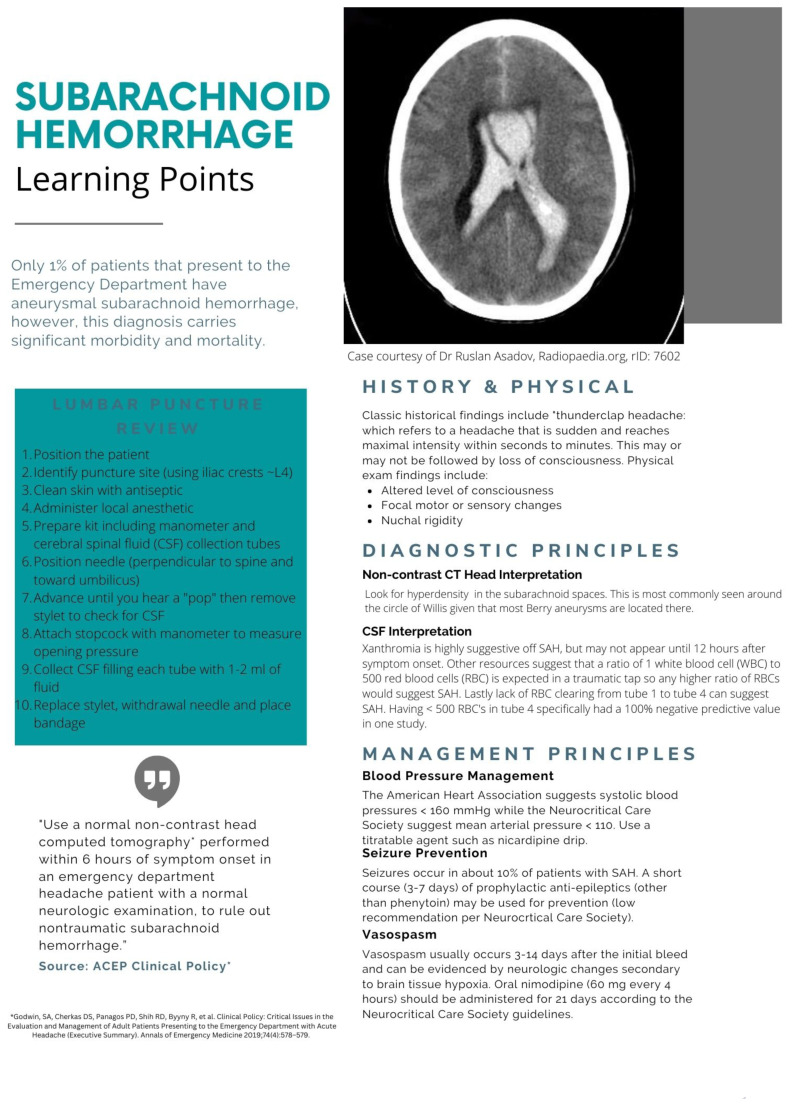


**Table t12-jetem-8-3-s34:** 

**Critical Actions:** Obtain comprehensive history, including high-risk features related to syncope and headaches Perform full neurologic exam Obtain blood glucose Obtain a non-contrast CT scan of the head Perform lumbar puncture Correctly interpret CSF results Lower blood pressure with IV agent, stating mean arterial pressure (MAP) goals to the nurse Admit to the Neurology Critical Care	0:00

**Table t13-jetem-8-3-s34:** 

	Milestone	Did not achieve level 1	Level 1	Level 2	Level 3
1	**Emergency Stabilization (PC1)**	□ Did not achieve Level 1	□ Recognizes abnormal vital signs	□ Recognizes an unstable patient, requiring intervention Performs primary assessment Discerns data to formulate a diagnostic impression/plan	□ Manages and prioritizes critical actions in a critically ill patient Reassesses after implementing a stabilizing intervention
2	**Performance of focused history and physical (PC2)**	□ Did not achieve Level 1	□ Performs a reliable, comprehensive history and physical exam	□ Performs and communicates a focused history and physical exam based on chief complaint and urgent issues	□ Prioritizes essential components of history and physical exam given dynamic circumstances
3	**Diagnostic studies (PC3)**	□ Did not achieve Level 1	□ Determines the necessity of diagnostic studies	□ Orders appropriate diagnostic studies. Performs appropriate bedside diagnostic studies/procedures	□ Prioritizes essential testing Interprets results of diagnostic studies Reviews risks, benefits, contraindications, and alternatives to a diagnostic study or procedure
4	**Diagnosis (PC4)**	□ Did not achieve Level 1	□ Considers a list of potential diagnoses	□ Considers an appropriate list of potential diagnosis May or may not make correct diagnosis	□ Makes the appropriate diagnosis Considers other potential diagnoses, avoiding premature closure
5	**Pharmacotherapy (PC5)**	□ Did not achieve Level 1	□ Asks patient for drug allergies	□ Selects an medication for therapeutic intervention, consider potential adverse effects	□ Selects the most appropriate medication and understands mechanism of action, effect, and potential side effects Considers and recognizes drug-drug interactions
6	**Observation and reassessment (PC6)**	□ Did not achieve Level 1	□ Reevaluates patient at least one time during case	□ Reevaluates patient after most therapeutic interventions	□ Consistently evaluates the effectiveness of therapies at appropriate intervals
7	**Disposition (PC7)**	□ Did not achieve Level 1	□ Appropriately selects whether to admit or discharge the patient	□ Appropriately selects whether to admit or discharge Involves the expertise of some of the appropriate specialists	□ Educates the patient appropriately about their disposition Assigns patient to an appropriate level of care (ICU/Tele/Floor) Involves expertise of all appropriate specialists
9	**General Approach to Procedures (PC9)**	□ Did not achieve Level 1	□ Identifies pertinent anatomy and physiology for a procedure Uses appropriate Universal Precautions	□ Obtains informed consent Knows indications, contraindications, anatomic landmarks, equipment, anesthetic and procedural technique, and potential complications for common ED procedures	□ Determines a back-up strategy if initial attempts are unsuccessful Correctly interprets results of diagnostic procedure
20	**Professional Values (PROF1)**	□ Did not achieve Level 1	□ Demonstrates caring, honest behavior	□ Exhibits compassion, respect, sensitivity and responsiveness	□ Develops alternative care plans when patients’ personal beliefs and decisions preclude standard care
22	**Patient centered communication (ICS1)**	□ Did not achieve level 1	□ Establishes rapport and demonstrates empathy to patient (and family) Listens effectively	Elicits patient’s reason for seeking health care	□ Manages patient expectations in a manner that minimizes potential for stress, conflict, and misunderstanding. Effectively communicates with vulnerable populations, (at risk patients and families)
23	**Team management (ICS2)**	□ Did not achieve level 1	□ Recognizes other members of the patient care team during case (nurse, techs)	□ Communicates pertinent information to other healthcare colleagues	□ Communicates a clear, succinct, and appropriate handoff with specialists and other colleagues Communicates effectively with ancillary staff

## INSTRUCTOR MATERIALS

**Case Title:** Headache Over Heels: CT Negative Subarachnoid Hemorrhage

**Case Description & Diagnosis (short synopsis):** This case involves a middle-aged male who experiences syncope secondary to subarachnoid hemorrhage. History and physical exams are significant for headache with maximum intensity at the onset with ongoing neck pain and stiffness. Unfortunately, the patient presents over 6 hours after onset, and the CT head is unremarkable, forcing the learner to obtain a lumbar puncture to ultimately make the diagnosis.

**Equipment or Props Needed:**

- High fidelity simulation mannequin
- Monitors
- IV poles
- Lumbar puncture kit

**Confederates needed:**

**Stimulus Inventory:** [Table t3-jetem-8-3-s34]

**Background and brief information:** You are working in a busy academic emergency department. You walk into the room to see a patient that arrived via private vehicle with a chief complaint of “syncope.”

**Initial presentation:** The patient is a 55-year-old male presenting after a syncopal episode. The patient states that he was out doing exertional yard work, including lifting heavy bags. His wife found him a few minutes later, lying in the grass. He denies any chest pain, shortness of breath, or tunnel vision prior to losing consciousness. He does endorse maybe feeling lightheaded and notes he had a severe headache prior to falling. He went inside out of the heat and started drinking water. He figured it was just dehydration or heat stroke but was still having headache, neck pain, and nausea, so he decided to come to the emergency department to be evaluated. He denies any vomiting.

**How the scene unfolds:** The learner should start by placing the patient on cardiac and pulse oximetry monitoring, revealing hypertension with normal heart rate, respiratory rate, and oxygen saturation. The learner should then obtain additional history. If asked more questions about his headache, the patient says he has never had a headache like this. He says it was the worst headache of his life but is somewhat better now. If asked about timing, the patient says the incident happened about seven hours ago (he presented four hours after the event and was in the waiting room for three hours). If asked further review of systems questions, the patient denies hematemesis or melena, tongue biting or urinary incontinence, confusion after falling, or a history of seizures. In addition, he denies any recent medication changes. The learner should then perform a physical exam that reveals an uncomfortable but non-toxic male with normal orientation and cranial nerves exam but nuchal rigidity and reduced neck flexion. The learner should then order diagnostic testing to include at least POC glucose, CBC, CMP, ECG, and CT head. Other optional testing may include troponin, d-dimer, bedside cardiac ultrasound, and vital orthostatic signs. CT head should be interpreted as negative for the acute intracranial process, but the learner should recognize that they cannot adequately rule out SAH based on a negative CT head at over 6 hours after symptom onset, and LP should be performed.

If supplies are available, the learner can perform a lumbar puncture on a mannequin. The learner should then diagnose SAH based on the interpretation of CSF results. Once the diagnosis is made, the learner should recheck the patient’s blood pressure, recognize that the systolic blood pressure is higher than the goal of 140–160, and initiate appropriate therapy (nicardipine or labetalol). The learner may choose to treat the patient’s pain at this time as well. Lastly, the learner should consult the Neurology Critical Care team for admission.

The learner should start by assessing vital signs. The patient is hypertensive but otherwise stable, not requiring immediate intervention, so the learner should then go on to obtain a full history and physical examination. Physical examination should specifically include a neurologic examination. If the learner fails to ask about headache or neck pain or fails to perform a neurologic examination, the facilitator should complain of headache and prompt the learner to assess neurologic function. Diagnostic studies should then be ordered, including blood glucose, ECG, and non-contrast CT scan of the head at a minimum. If the learner fails to order a CT head, the facilitator should ask, “Are there any tests you would like to order to work up the patient’s headache?” or, “What do you make of the patient’s headache?” The learner will then receive lab and CT head results (as well as other testing results they ordered). After interpreting the CT head as negative, the learner should suggest a lumbar puncture to further investigate for SAH. If the learner is unfamiliar with this algorithm, they may need to be prompted, “If you are worried about a SAH, but CT head is negative, is there another study you can pursue?” A lumbar puncture can either be performed on a mannequin if able or verbally explained in a step-wise fashion. The learner will be given CSF results that are consistent with SAH. They should then reassess vitals and recognize that the patient is still hypertensive with systolic blood pressure above the goal of less than 140–160. They should then order a titratable intravenous antihypertensive agent. If they do not order medications, the facilitator may ask them what the blood pressure goals are in SAH. Lastly, the learner should contact the Neurology Critical Care team for definitive disposition.

**Critical actions:**

1. Obtain comprehensive history, including high-risk features related to syncope and headaches
2. Perform full neurologic exam
3. Obtain blood glucose
4. Obtain a non-contrast CT scan of the head
5. Perform lumbar puncture
6. Correctly interpret CSF results
7. Lower blood pressure with IV agent, stating mean arterial pressure (MAP) goals to the nurse
8. Admit to the Neurology Critical Care

**Case Title:** Headache Over Heels: CT Negative Subarachnoid Hemorrhage

**Chief Complaint:** Syncope[Table t4-jetem-8-3-s34]

**General Appearance:** Uncomfortable but non-toxic appearing

**Primary Survey:**

- **Airway:** patent
- **Breathing:** non-labored, clear, bilateral breath sounds
- **Circulation:** Normal rate, regular rhythm, palpable bilateral radial, and dorsalis pedis pulses

**History:**

- **History of present illness:** The patient is a 55-year-old male presenting after a syncopal episode. The patient states that he was out doing yard work, including lifting heavy bags. His wife found him a few minutes later, lying in the grass. He denies any chest pain, shortness of breath, or tunnel vision prior to losing consciousness. He does endorse maybe feeling lightheaded and notes he had a severe headache prior to falling. He says he has never had a headache like this before, but it is definitely better now. He describes the headache as bilateral posterior sharp pains that radiate to the neck. He went inside out of the heat and started drinking water. He figured it was just dehydration or heat stroke but was still having headaches, neck pain, and nausea, so he decided to come in. He says he never vomited. The incident happened about seven hours ago (he arrived at the ED four hours after the event and was in the waiting room for three hours). Otherwise, he has been feeling well with no recent symptoms. He denies recent changes to medications. He denies recent hematemesis or melena. The event was unwitnessed, but he denies tongue biting or urinary incontinence. He denies any confusion after the event. He has no history of seizures.
- **Past medical history:** Hypertension, hyperlipidemia, type 2 diabetes
- **Past surgical history:** none
- **Medications:** amlodipine, atorvastatin, metformin
- **Allergies:** penicillin (hives)
- **Social history:** ½ ppd smoker for 30 years, drinks 4–5 beers per week, no illicit drug use
- **Family history:** Paternal uncle died of an aneurysm rupture in his 50’s

**Secondary Survey/Physical Examination:**

- **General appearance:** uncomfortable but non-toxic appearing
- **HEENT:**
  ○ **Head:** within normal limits (wnl)
  ○ **Eyes:** wnl
  ○ **Ears:** wnl
  ○ **Nose:** wnl
  ○ **Throat:** wnl
- **Neck:** Nuchal rigidity with reduced neck flexion, no JVD, carotid pulses auscultated bilaterally without bruit
- **Heart:** wnl
- **Lungs:** wnl
- **Abdominal/GI:** wnl
- **Genitourinary:** wnl
- **Rectal:** wnl
- **Extremities:** wnl
- **Neuro:** alert, oriented x4, no dysarthria, no facial droop, CN II–XII grossly intact, sensation to light touch diminished in the right upper extremity compared to the left upper extremity, normal sensation to light touch in bilateral lower extremities, 5/5 left shoulder abduction, forearm extension, forearm flexion and hand grip, 4/5 right shoulder abduction, forearm extension, forearm flexion, and hand grip, 5/5 strength in all muscle groups of the lower extremities, no dysmetria on bilateral finger-to-nose and heel-to-shin, normal gait observed
- **Skin:** wnl
- **Lymph:** wnl
- **Psych:** wnl[Table t5-jetem-8-3-s34][Table t6-jetem-8-3-s34][Table t7-jetem-8-3-s34][Table t8-jetem-8-3-s34][Table t9-jetem-8-3-s34]

*Chest X-ray*

Gaillard F. Normal chest x-ray. In: Radiopaedia. Accessed March 25, 2023. CC BY-NC-SA 3.0 At: https://doi.org/10.53347/rID-8304[Fig f1-jetem-8-3-s34]

*Electrocardiogram*

Burns E and Buttner R. Normal ECG. In: Life in the Fast Lane. Published March 11, 2021. Accessed November 20, 2021. CC BY-NC-SA 4.0. At: https://litfl.com/normal-sinus-rhythm-ecg-library/[Fig f2-jetem-8-3-s34]

*Computed Tomography Head*

Cuete, D. Normal CT brain. In: Radiopaedia. Published July 7, 2013. Accessed November 20, 2021. At: https://radiopaedia.org/cases/23768?lang=us.[Fig f3-jetem-8-3-s34][Table t10-jetem-8-3-s34]

## OPERATOR MATERIALS

[Table t11-jetem-8-3-s34]

**Diagnosis:**

Subarachnoid Hemorrhage

**Disposition:**

Admitted to the neurology ICU.

## DEBRIEFING AND EVALUATION PEARLS

### Headache Over Heels: CT Negative Subarachnoid Hemorrhage Chief

#### Syncope

■ Differential Diagnosis of syncope is broad and includes:^9^
  ○ Cardiogenic causes
    ■ Arrhythmia
    ■ Structural/Obstructive
      - Valvular Disease
      - Aortic Dissection
      - Myocardial Infarction
      - Congestive Heart Failure
      - Hypertrophic Cardiomyopathy
      - Pulmonary Embolism
      - Pericardial Tamponade
      - Myxoma
      - Pulmonary Hypertension
      - Pacemaker Malfunction
  ○ Neurogenic causes
    ■ Stroke
    ■ SAH
    ■ Seizure
  ○ Vasovagal
  ○ Orthostatic Causes
    ■ Volume Depletion
    ■ Medication-related
    ■ Autonomic Dysfunction
  ○ Other
    ■ Ectopic Pregnancy
    ■ Rupture Abdominal Aortic Aneurysm
    ■ Hypoglycemia
    ■ Toxic Ingestion

#### SAH

■ History and physical exam findings:
  ○ Patients may report sudden, severe headache with maximum intensity shortly after onset
  ○ History may also include syncope, nausea and vomiting, neck pain or stiffness, and neurologic complaints
  ○ On examination, patients may have an altered level of consciousness, focal neurologic deficits, or nuchal rigidity/limited neck flexion
■ Imaging
  ○ SAH appears as blood in the subarachnoid spaces (ventricles, cisterns, sulci) on CT head imaging
  ○ CT Head without contrast sensitivity for SAH depends on the concentration of hemoglobin which becomes hemosiderin over time
    ■ ACEP Clinical Policy: “Use a normal non-contrast head computed tomography^*^ performed within 6 hours of symptom onset in an emergency department headache patient with a normal neurologic examination to rule out nontraumatic subarachnoid hemorrhage.”^4^
  ○ Per ACEP clinical policy, CTA is a reasonable alternative to LP if CT head is negative for SAH^4^
    ■ Cons include exposure to radiation as well as the potential to diagnose asymptomatic aneurysms^4^
■ Lumbar Puncture
  ○ Indicated if high clinical suspicion for SAH but CT head is negative, and patient is presenting 6 hours or more after symptom onset because CT sensitivity starts to decline at the 6-hour mark^4^
  ○ Results will include RBCs that do not decrease across tubes one through four
  ○ Xanthochromia (or bilirubin in the CSF) may also be present secondary to breakdown of RBCs^6^
■ Blood Pressure Management
  ○ The American Heart Association suggests a goal of systolic blood pressure < 160 mmHg^8^
  ○ The Neurocritical Care Society suggests a goal of mean arterial pressure < 110 mmHg^3^
  ○ Treat pain first. Consider using fentanyl or morphine.
  ○ In terms of a specific antihypertensive agent, the American Heart Association just recommends a “titratable agent” such as nicardipine or labetalol drip^8^
■ Pain control
  ○ Treat pain first. Consider using fentanyl or morphine.
■ Glycemic control
  ○ Avoid hypoglycemia < 80 and avoid hyperglycemia > 200, according to the Neurocritical Care Society^3^
■ Airway
  ○ If you need to intubate this patient, consider efforts to decrease intracranial pressure (ICP), such as pre-treatment with fentanyl^3^
  ○ If elevated ICP is suspected, hyperventilation can be used as a temporary measure until definitive management with external drainage is possible because hyperventilation can cause cerebral vasoconstriction and thus cerebral hypoperfusion if used long-term^6^
■ Seizure prophylaxis
  ○ Seizures occur in about 10% of patients; current recommendations are that all SAH patients are treated with Keppra for 3 to 7 days^3^
  ○ The Neurocritical Care Guideline specifically suggests against phenytoin use because some studies have shown worse long-term outcomes in SAH patients treated with phenytoin^3^
■ Disposition:
  ○ The patient should be admitted to the Intensive Care Unit, or if available Neurology Intensive Care Unit with neurosurgery consultation for surgical clipping versus endovascular coiling as soon as possible. If neurosurgery is not available at the hospital, the patient should be transferred for a higher level of care to a hospital that does have neurosurgery.
    ■ Less time for definitive treatment is associated with less risk of rebleeding, according to the American Heart Association^8^
■ Vasospasm –
  ○ Usually occurs 3 to 14 days after the inciting event^3^
  ○ It is a significant cause of morbidity if patients survive the initial incident^3^
  ○ The Modified Fischer Scale helps estimate the risk of vasospasm
  ○ Vasospasm can cause tissue ischemia, so the patients present with sudden worsening of their neurologic examination^3^
  ○ Use calcium channel blockers to try to prevent vasospasm (nimodipine 60 mg every 4 hours for 21 days), but this may be reduced in patients with borderline hypotension (more frequent lower doses can be used instead)^3^

**Wrap Up:** Wrap up worksheet is attached.[Fig f4-jetem-8-3-s34]

## SIMULATION ASSESSMENT

*Headache Over Heels: CT Negative Subarachnoid Hemorrhage*

Learner: _________________________________________

***Assessment Timeline***

This timeline is to help observers assess their learners. It allows observer to make notes on when learners performed various tasks, which can help guide debriefing discussion.[Table t12-jetem-8-3-s34]

**Critical Actions:**

□ Obtain comprehensive history, including high-risk features related to syncope and headaches
□ Perform full neurologic exam
□ Obtain blood glucose
□ Obtain a non-contrast CT scan of the head
□ Perform lumbar puncture
□ Correctly interpret CSF results
□ Lower blood pressure with IV agent, stating mean arterial pressure (MAP) goals to the nurse
□ Admit to the Neurology Critical Care

**Summative and formative comments:**

**Milestones assessment:** [Table t13-jetem-8-3-s34]

## References

[1] Hine J Marcolini E Ashenburg N Aneurysmal Subarachnoid Hemorrhage EMRAP Updated: September 17th, 2020 Accessed June 16, 2022 Available at: https://www.emrap.org/corependium/chapter/recTI59VW0TPBpesx/Aneurysmal-Subarachnoid-Hemorrhage#h.tfpbnznfnawt

[2] Long B Koyfman A Runyon MS Subarachnoid Hemorrhage: Updates in Diagnosis and Management Emerg Med Clin North Am 2017 35 4 803 824 28987430 10.1016/j.emc.2017.07.001

[3] Diringer MN Bleck TP Claude Hemphill J 3rd Critical care management of patients following aneurysmal subarachnoid hemorrhage: recommendations from the Neurocritical Care Society’s Multidisciplinary Consensus Conference Neurocrit Care 2011 15 2 211 240 10.1007/s12028-011-9605-9 21773873

[4] Godwin SA Cherkas DS Panagos PD Clinical Policy: Critical Issues in the Evaluation and Management of Adult Patients Presenting to the Emergency Department with Acute Headache (Executive Summary) Ann Emerg Med 2019 74 4 578 579 10.1016/j.annemergmed.2019.07.009 31543134

[5] Perry JJ Alyahya B Sivilotti ML Differentiation between traumatic tap and aneurysmal subarachnoid hemorrhage: prospective cohort study BMJ 2015 350 h568 Published 2015, Feb 18. At 10.1136/bmj.h568 25694274 PMC4353280

[6] Farkas J Subarachnoid Hemorrhage (SAH) EM Crit Accessed June 16, 2022 Available at: https://emcrit.org/ibcc/sah/

[7] Gorchynski J Oman J Newton T Interpretation of traumatic lumbar punctures in the setting of possible subarachnoid hemorrhage: who can be safely discharged? Cal J Emerg Med 2007 8 1 3 7 20440386 PMC2859734

[8] Connolly ES Rabinstein AA Carhuapoma JR Guidelines for the management of aneurysmal subarachnoid hemorrhage: a guideline for healthcare professionals from the American Heart Association/American Stroke Association Stroke 2012 43 6 1711 37 22556195 10.1161/STR.0b013e3182587839

[9] Ross D Celedon M Swartz J Syncope WikEM June 01 2022 Accessed March 25, 2023 At: https://wikem.org/wiki/Syncope

