# Supplementary figures and images for: Headache Over Heels: CT Negative Subarachnoid Hemorrhage
Source: J Educ Teach Emerg Med. 2023 Jul 31;8(3):S34–58. doi: 10.21980/J8ND2C (PMC10414981; doi:10.21980/J8ND2C)

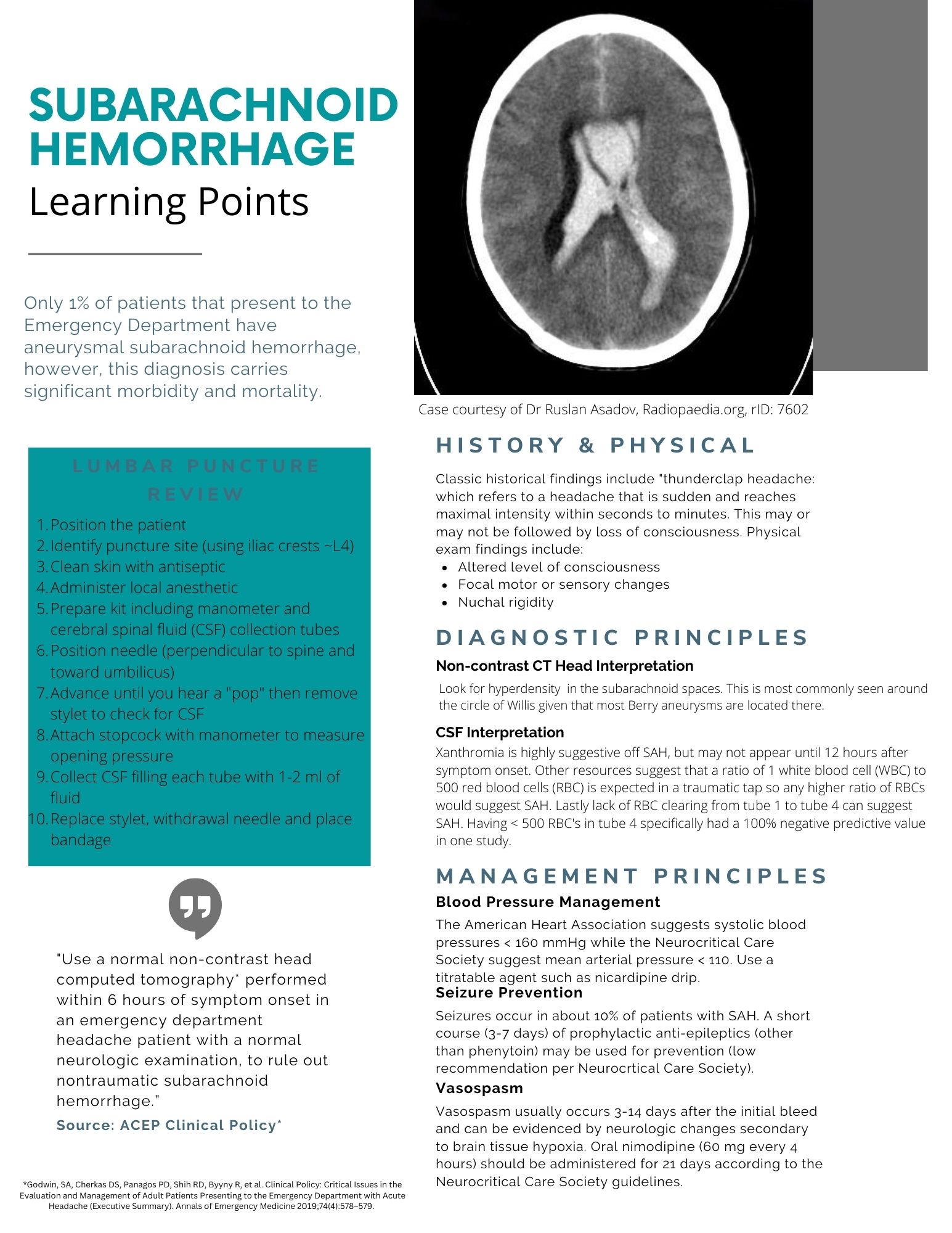

Supplement: Supplementary file 1 [file JETem-8-3-S34-supp1.jpg]
